# SMKD: Selective Mutual Knowledge Distillation

**DOI:** 10.1109/IJCNN54540.2023.10191991

**Published:** 2023-06-18

**Authors:** Ziyun Li, Xinshao Wang, Neil M. Robertson, David A. Clifton, Christoph Meinel, Haojin Yang

**Affiliations:** Hasso Plattner Institute, University of Potsdam, Potsdam, Germany; University of Oxford, Oxford, United Kingdom; Queen’s University Belfast, Belfast, United Kingdom; University of Oxford, Oxford, United Kingdom; Hasso Plattner Institute, University of Potsdam, Potsdam, Germany; Hasso Plattner Institute, University of Potsdam, Potsdam, Germany

## Abstract

Mutual knowledge distillation (MKD) is a technique used to transfer knowledge between multiple models in a collaborative manner. However, it is important to note that not all knowledge is accurate or reliable, particularly under challenging conditions such as label noise, which can lead to models that memorize undesired information. This problem can be addressed by improving the reliability of the knowledge source, as well as selectively selecting reliable knowledge for distillation. While making a model more reliable is a widely studied topic, selective MKD has received less attention. To address this, we propose a new framework called selective mutual knowledge distillation (SMKD). The key component of SMKD is a generic knowledge selection formulation, which allows for either static or progressive selection thresholds. Additionally, SMKD covers two special cases: using no knowledge and using all knowledge, resulting in a unified MKD framework. We present extensive experimental results to demonstrate the effectiveness of SMKD and justify its design.

## I Introduction

*“What knowledge to be selected for distillation”* is an essential question of mutual knowledge distillation (MKD) but has received little attention. In this work, we study it for two reasons: (i) Existing MKD methods treat all knowledge of a deep model equally, i.e., all knowledge is distilled into another model without selection. (ii) There are two contradictory findings of label smoothing(LS). One is that in clean scenarios, when a network (a teacher) is trained with LS, distilling its knowledge into another model (a student) is much less effective [1]. Another finding is that in noisy scenarios, LS improves both teacher and student. In their contradictory studies, they only focus on the knowledge source, e.g., how a source (teacher) model is trained. There was no study on the knowledge selection, which could be a key, as empirically indicated in Table I. This research question can also be expressed as:

Should all knowledge or partial knowledge of a model be distilled into another model?

In clean scenarios, the knowledge source is generally reliable. Thus, simply distilling all knowledge is reasonable, and it has widespread use in existing KD works. However, in label-noise scenarios^1^, the knowledge source is less reliable. The distilled incorrect knowledge would mislead the learning rather than help. Therefore, it is vital to note “not all knowledge is created equal” and identify “what knowledge could be distilled?”. We work on this problem from two aspects: (i) *making the knowledge source more reliable*. (ii) *selecting the certain knowledge to distill*. For the first aspect, many algorithms have been proposed, e.g., Tf-KD [3] and ProSelfLC [2]. For simplicity, we exploit them and focus more on the second aspect: *selective knowledge distillation*.

**Fig. 1:**
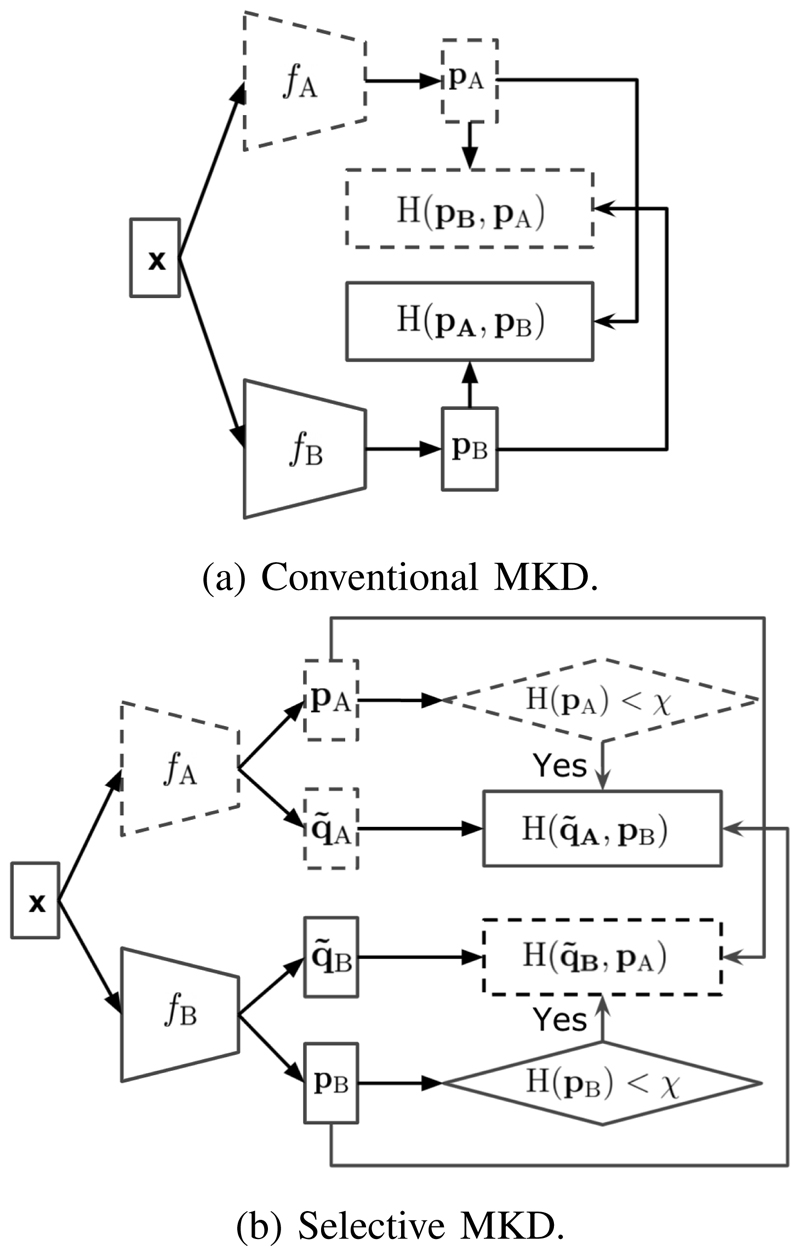
Comparison of conventional MKD and our SMKD. Dotted frames represent components from model A and solid frames represent components from model B. **p**_A_ and **p**_B_ are predictions from mode A and model B, respectively. In (b), ${\tilde{\text{q}}}_{\text{A}}$ and ${\tilde{\text{q}}}_{\text{B}}$ represent the refined labels by a self distillation method, and *χ* is the threshold to decide whether the prediction is confident enough or not. H(**p**) denotes the entropy of **p**, and H(**q**, **p**) is the cross entropy loss between **q** and **p**.

To explore the knowledge selection problem, we design a selective mutual knowledge distillation (SMKD) framework, which is shown in Figure 1. We propose to only distill confident knowledge. Specifically, we design a generic knowledge selection formulation, so that we can either fix the knowledge selection threshold *(SMKD-Static, shortened as SMKD-S)* or change it progressively as the training progresses *(SMKD-Progressive, abbreviated as SMKD-P)*. In SMKD-P, we leverage the training time to adjust how much knowledge would be selected dynamically considering that a model’s knowledge improves along with time. SMKD-P performs slightly better than SMKD-S, according to our empirical studies, e.g., Table I.

We summarise our contributions as follows:

- We study what knowledge to be selected for distillation in MKD. Correspondingly, we propose a generic knowledge selection formulation, which covers the variants of zero-knowledge, all knowledge, SMKD-S, and SMKD-P.
- Thorough studies on the models’ learning curves, knowledge selection criterion’s settings, and hyperparameters justify the rationale of our selective MKD design and its effectiveness.
- Our proposed SMKD outperforms previous MKD algorithms in the presence of label noise.

## II Background

We give an introduction about knowledge distillation and learning with label noise methods.

### A Notations

For a multi-class classification problem, **x** is a data point, and **q** ∈ **R**^*C*^ is its annotated label distribution, also seen as annotated knowledge. *C* is the number of training classes. In the traditional practice, **q** is a one-hot representation, a.k.a., hard label. Mathematically, **q**(*j* |**x**) = 1 only if *j* = *y*, and 0 otherwise. Here, *y* denotes the semantic class of **x**. *f* is a deep neural network that predicts the probabilities of **x** being different training classes. We denote them using a vector **p** ∈ **R**^C^, which can be seen as a model’s self knowledge.

### B Knowledge Distillation

KD is an effective method for distilling the knowledge of complex ensembles or a cumbersome model (usually named teacher models) to a small model (usually named a student) [6], [7]. Recently, many deep KD variants have been proposed, e.g., self knowledge distillation (Self KD) which trains a single learner and leverages its own knowledge [2], [3], MKD with knowledge transfer between two learners [8], [9], [10], ensemble-based KD methods [11], [12], and born-again networks with knowledge distilling from multiple student generations [13]. Since we focus on training two learners, Teacher→Student KD (T2S KD) and MKD are more relevant, we briefly present them as follows.

T2S KD [7] transfers knowledge from a teacher model to a student model and be formulated as:

(1) $$L_{\text{T}2\text{SKD}}\left(\text{q},\text{p},{\text{p}}_{t}\right)=(1-\epsilon )\text{H}(\text{q},\text{p})+\epsilon {\text{D}}_{\text{KL}}\left({\text{p}}_{t},\text{p}\right),$$

where **q** is the given one-hot label, **p** is the predicted distribution by a student model and **p**_*t*_ is the output of a teacher model. H(**q**, **p**) represents the cross entropy loss between target **q** and prediction **p**. D_KL_ (**p**_*t*_, **p**) denotes the Kullback–Leibler (KL) divergence of **p**_*t*_ from **p**.

MKD [8] trains two models A and B, making them learn from each other as follows:

(2) $$\begin{array}{l} L_{\text{A}}\left(\text{q},{\text{p}}_{\text{A}},{\text{p}}_{\text{B}}\right)=(1-\epsilon )\text{H}\left(\text{q},{\text{p}}_{\text{A}}\right)+\epsilon {\text{D}}_{\text{KL}}\left({\text{p}}_{\text{B}},{\text{p}}_{\text{A}}\right)\\ L_{\text{B}}\left(\text{q},{\text{p}}_{\text{B}},{\text{p}}_{\text{A}}\right)=(1-\epsilon )\text{H}\left(\text{q},{\text{p}}_{\text{B}}\right)+\epsilon {\text{D}}_{\text{KL}}\left({\text{p}}_{\text{A}},{\text{p}}_{\text{B}}\right)\\ L_{\text{MKD}}=L_{\text{A}}\left(\text{q},{\text{p}}_{\text{A}},{\text{p}}_{\text{B}}\right)+L_{\text{B}}\left(\text{q},{\text{p}}_{\text{B}},{\text{p}}_{\text{A}}\right). \end{array}$$

Other ensemble-based and feature-map-based KD methods, e.g., knowledge distillation via collaborative learning (KDCL) [11] treats all models as students, while the teacher model is an ensemble of all students. Peer collaborative learning (PCL)[12] assembles multiple subnetworks as a teacher model. FFL[14] integrates feature representation of multiple models and AFD[15] transfers prediction and feature-map knowledge together.

### C Learning with Label Noise

We compare recent methods for learning with noisy labels, including: selecting confident samples: Co-teaching [16] and Co-teaching+ [17] maintain two identical networks simultaneously and transfer small-loss instances to the peer model. Providing a curriculum: MentorNet [18] provides a curriculum for StudentNet to focus on the examples with likely-correct labels. Correcting training loss: Joint [19] and Forward [20] correct training loss through the calculation of the noise transition matrix. Sample reweighting: T-revision [21] reweights samples based on their significance. Designing robust loss function: DMI [22] introduces an information-theoretic loss function, and APL [23] combines two robust loss functions that mutually boost each other. Early stopping: CDR [24] reduces the side effect of noisy labels before early stopping. Label modification, which is an important technique related to our topic, will be discussed in more detail in the following subsection.

### D Label Modification

Label modification is used to improve the accuracy of a model by correcting the labels of the training data. This can be done by identifying and correcting errors in the labels, or by actively relabeling a subset of the data to improve the overall performance of the model. As mentioned in [3], the learning target modification is to replace a one-hot label representation by its convex combination with a predicted distribution $\tilde{\text{p}}$:

(3) $$\tilde{\text{q}}=(1-\epsilon )\text{q}+\epsilon \tilde{\text{p}}.$$

*ϵ* measures how much we trust the prediction, and it can be fixed in Label smoothing(LS) [25], Confidence penalty (CP) [26], Boot-soft [27], Joint-soft [19], or adaptive by training time e.g., [2] and [3]. In Section IV-B, we also present an alternative label modification approach, MyLC, in which *ϵ* is updated by model confidence. $\tilde{\text{p}}$ can originate from various sources, such as uniform distributions, a current model, a model that has been pretrained, etc. By adding a uniform distribution, for example, LS reduces the confidence in annotated label. CP reduces the credibility of annotated labels by penalizing high confidence predictions. By incorporating a related prediction, Boot-soft, Tf-KD, ProselfLC and MyLC refine the learning target.

## III Method

We design a generic knowledge selection formulation that unifies zero knowledge, all knowledge, and partial knowledge selection in a static and progressive fashion (SMKD-S and SMKD-P). The pseudocode of the algorithm is provided at the end of Section III.

### A Learning Objectives

To distill model B’s confident knowledge into model A, we optimise A’s predictions towards B’s confident predictions:

(4) $$L_{\text{B}2\text{A}}=\{\begin{array}{ll} \text{H}\left({\tilde{\text{q}}}_{\text{B}},{\text{p}}_{\text{A}}\right), & \text{H}\left({\text{p}}_{\text{B}}\right)<\chi ,\\ 0, & \text{H}\left({\text{p}}_{\text{B}}\right)\geq \chi . \end{array}$$

We use the entropy H(**p**_B_) to measure the confidence of **p**_B_. Low entropy indicates high confidence, and vice versa [26], [25], [7], [28]. *χ* is a threshold to decide whether a label prediction is confident enough or not. Specifically, only when H(**p**_B_) < *χ*, the model B’s knowledge w.r.t. **x** is confident enough. ${\tilde{\text{q}}}_{\text{B}}$ is the model B’s learning target, which can be generated by a self label modification method as it is more reliable. Note that instead of directly distilling confident predictions **p**_B_, we transfer targets (refined labels) ${\tilde{\text{q}}}_{\text{B}}$ that produce confident predictions.

Analogously, we distill model A’s confident knowledge into model B:

(5) $$L_{\text{A}2\text{B}}=\{\begin{array}{ll} \text{H}\left({\tilde{\text{q}}}_{\text{A}},{\text{p}}_{\text{B}}\right), & \text{H}\left({\text{p}}_{\text{A}}\right)<\chi ,\\ 0, & \text{H}\left({\text{p}}_{\text{A}}\right)\geq \chi . \end{array}$$

The final loss functions for models A and B are:

$$\begin{array}{l} L_{\text{A}}=L_{{\text{A}}_{\text{selfKD }}}+L_{\text{B}2\text{A}}\\ \;=\{\begin{array}{ll} \text{H}\left({\tilde{\text{q}}}_{\text{A}},{\text{p}}_{\text{A}}\right)+\text{H}\left({\tilde{\text{q}}}_{\text{B}},{\text{p}}_{\text{A}}\right), & \text{H}\left({\text{p}}_{\text{B}}\right)<\chi ;\\ \text{H}\left({\tilde{\text{q}}}_{\text{A}},{\text{p}}_{\text{A}}\right), & \text{H}\left({\text{p}}_{\text{B}}\right)\geq \chi ; \end{array} \end{array}$$

$$\begin{array}{l} L_{\text{B}}\;=L_{{\text{B}}_{\text{selfKD }}}+L_{\text{A}2\text{B}}\\ =\{\begin{array}{ll} \text{H}\left({\tilde{\text{q}}}_{\text{B}},{\text{p}}_{\text{B}}\right)+\text{H}\left({\tilde{\text{q}}}_{\text{A}},{\text{p}}_{\text{B}}\right), & \text{H}\left({\text{p}}_{\text{A}}\right)<\chi ;\\ \text{H}\left({\tilde{\text{q}}}_{\text{B}},{\text{p}}_{\text{B}}\right), & \text{H}\left({\text{p}}_{\text{A}}\right)\geq \chi ; \end{array} \end{array}$$

### B A Generic Design for Knowledge Selection

As aforementioned, we use an entropy threshold *χ* to decide whether a piece of knowledge is certain enough or not. We design a generic formation for *χ* as follows:

(6) $$\chi =\frac{\text{H}(\text{u})}{\eta }*2h\left(\frac{t}{\text{Γ}}-0.5,b_{2}\right),$$

where *h*(·, ·) is a logistic function. **u** is a uniform distribution, thus H(**u**) is a constant. *t* and Γ denote the current epoch and the total number of epochs, respectively. For a wider unification, we make the design of Eq. (6) generic and flexible. Therefore, we use *η* to control the starting point. While *b*_2_ controls how the knowledge selection changes along with *t*. *χ* has two different modes:

- **Static (SMKD-S)**. The confidence threshold *χ* is a constant when *b*_2_ = 0. Concretely, $2h\left(\frac{t}{\text{Γ}}-0.5,0\right)=1\to \chi =\frac{\text{H}(\text{u})}{\eta }$. This mode covers two special cases:
  (i) One model’s all knowledge is distilled into the other when *η* ∈ (0,1] → *χ* ≥ H(**u**), which degrades to be the conventional MKD.
  (ii) Zero knowledge is distilled between two models when *η* ∈ {+∞, ℝ^–^} → *χ* ≤ 0.
- **Progressive (SMKD-P)**. When *b*_2_ ≠ 0, *χ* changes as the training progresses. To make it comprehensive, χ can be either increasing or decreasing at training:
  (i) If *b*_2_ > 0, *χ* increases as t increases. Since the knowledge selection criteria is relaxed, more knowledge will be transferred between the two models at the later learning phase.
  (ii) On the contrary, *χ* gradually decreases when setting *b*_2_ < 0. This only allows knowledge with higher confidence (lower entropy) to be distilled.

**Fig. 2:**
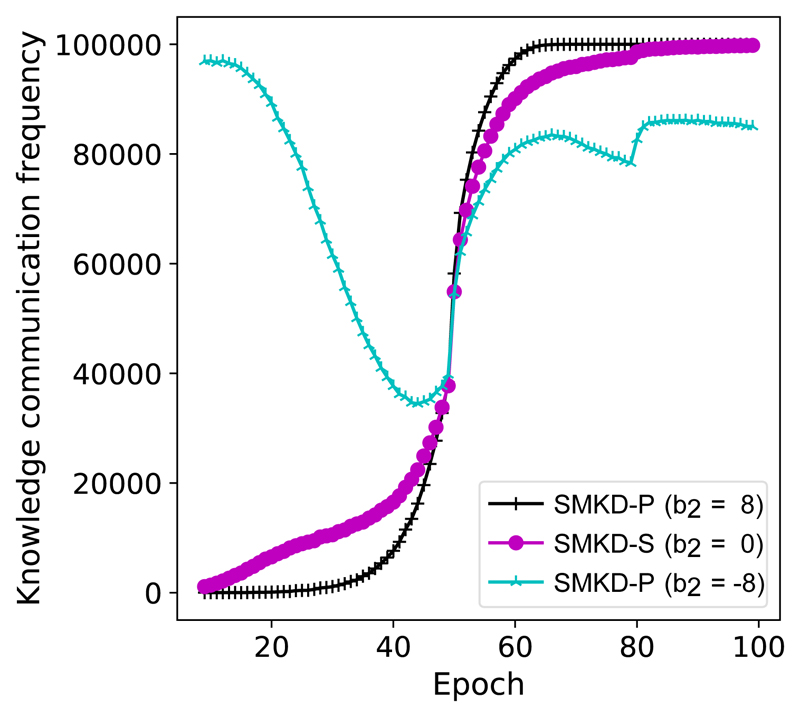
Knowledge communication frequency is measured by the sum of the number of distilled knowledge (training labels) from A to B and that from B to A. All experiments are done on CIFAR-100 with *η* = 2 under 40% symmetric noise. CIFAR-100 has 50,000 training examples in total and most of the training samples are exploited in the late training processing.

**Fig. 3:**
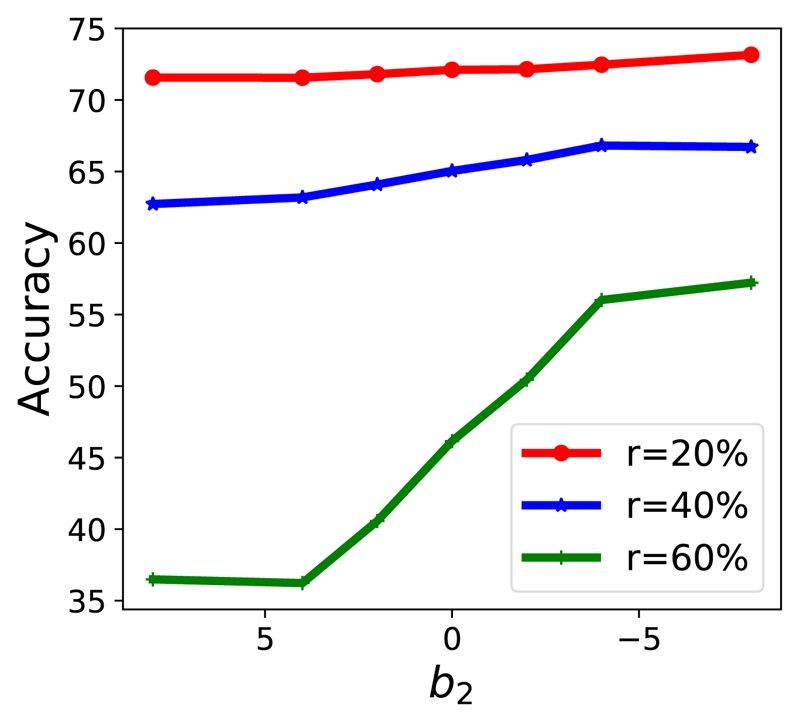
Under different noise rates. We fix *η* = 2.

**Fig. 4:**
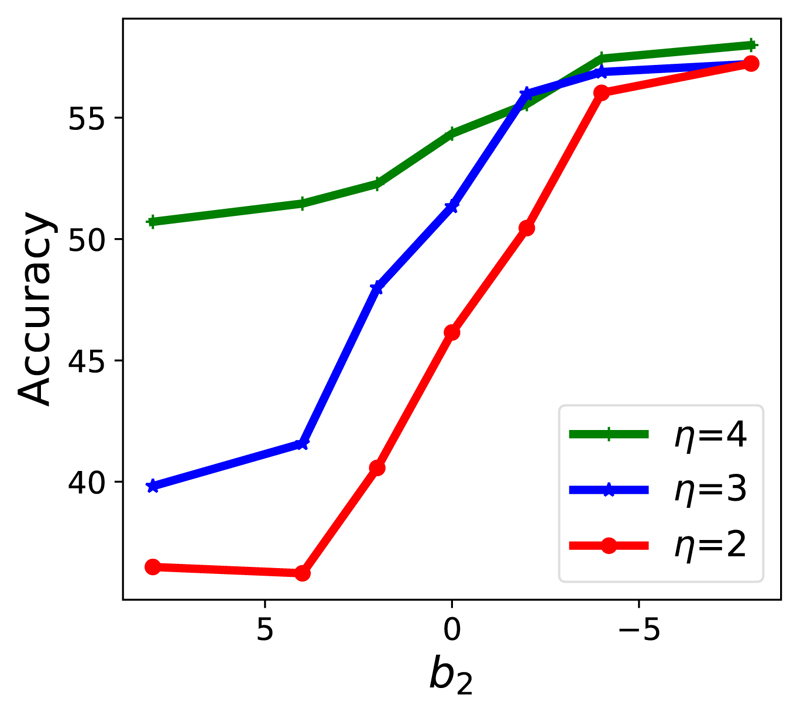
Under different *η*. Symmetric label noise rate *r* = 60%.

It is worth highlighting that compared to sample selection methods, such as [16], SMKD can correct the supervision in loss computation and optimisation stages when the supervision (label) is noisy. In other word, instead of discarding noisy samples, SMKD can correct supervision and distill reliable knowledge. Both models’ knowledge becomes more confident at the later stage even the knowledge selection criterion becomes stricter (i.e., *b*_2_ < 0). And we can clearly observe that almost all the training samples are distilled in the later training phase in Figure 2. In our empirical studies (e.g., Figure 3, Figure 4), in the noisy scenario, SMKD-P with *b*_2_ < 0 performs the best. Therefore, when comparing with prior relevant methods, we use SMKD-P with *b*_2_ < 0 by defaults.

## IV Experiments

In this section, we first demonstrate that SMKD is effective in robust learning against an adverse condition, i.e., label noise (Section IV-C). Then we empirically verify that SMKD, as a selective MKD, outperforms prior MKD approaches for training two models collaboratively no matter whether they are of the same architecture or not (Section IV-D). We subsequently present a comprehensive hyper-parameters analysis (Sections IV-E). Different network architectures are evaluated. For all experiments, we report the final results when the training terminates. For a more thorough comparison, we also provide an alternate self-training method called MyLC in IV-B.

### A Experimental Setup

#### a) Datasets and Data Augmentation

- CIFAR100 [29] has 50,000 training images and 10,000 test images of 100 classes. The image size is 32 × 32 × 3. Simple data augmentation is applied following [30], i.e., we pad 4 pixels on every side of the image and then randomly crop it with a size of 32×32.
- Food-101 [31] has 75,750 images of 101 classes. The training set contains real-world noisy labels. In the test set, there are 25,250 images with clean labels. For data augmentation, training images are randomly cropped with a size of 224 × 224.
- Webvision [18] has 2.4 million images crawled from the websites using the 1,000 concepts in ImageNet ILSVRC12 [32]. For data augmentation, we first resize the training images to 320 × 320 and then randomly cropped with a size of 299 × 299.

#### b) Implementation Details

We train on the CIFAR100, Food-101, and Webvision datasets using various architectures and settings. On CIFAR100, we use 90% of the training data (corrupted in synthetic cases) for training and 10% as a validation set to search for hyperparameters, and retrain the model on the entire dataset before reporting accuracy on the test data. We train on three architectures including ResNet34, ResNet50, and ShuffleNetV2, using an SGD optimizer with a momentum of 0.9, a weight decay of 5e-4, and a batch size of 128. On Food-101, we use 90% of the training data for training and 10% for validation, reporting accuracy on the clean test data. We train ResNet50 (initialized by a pretrained model on ImageNet) with a batch size of 32, and use an SGD optimizer with a momentum of 0.9 and a weight decay of 5e-4. On Webvision, we follow the “Mini” setting in [18], using the first 50 classes of the Google resized image subset as the training set and the same 50 classes of the ILSVRC12 validation set as the test set, training with the inception-resnet v2 architecture with a batch size of 32, and an SGD optimizer with a momentum of 0.9 and a weight decay of 5e-4.

### B MyLC: An Alternative for Label Modification

MyLC is designed for demonstrating the effectiveness and extensiveness of SMKD, which serves as an alternative to label modification methods. Note that MyLC is different from ProselfLC methods in terms of working principle. Furthermore, MyLC solves a significant drawback of ProselfLC that the model always has to be trained from scratch, since ProselfLC relies on training time. MyLC is obviously more suitable if we want to do fine-tuning or incremental learning tasks based on pretrained models. Specifically, without considering training time, MyLC defines the global model confidence according to a model’s predictive confidence w.r.t. all samples and is computed as follows:

(7) $$g(r)=h\left(r-\rho ,b_{1}\right),\;\text{ where }r=1-\frac{\sum_{i=1}^{n}\text{H}\left({\text{p}}_{i}\right)}{n*\text{H}(\text{u})}.$$

*h*(*λ*, *b*_1_) = 1/(1 + exp(–*λ* × *b*_1_) is a logistic function, where *b*_1_ is a hyperparameter for controlling the smoothness of *h*. This is widely used in semi-supervised learning[33], [34] and label noise learning [2]. *r* represents a model’s overall certainty of all examples. A higher *r* implies that a model is more reliable. Intuitively, if *r* is higher than a threshold *ρ*, we should assign more trust to the model. We simply set *ρ* = 0.5 in all our experiments. Consequently, Consequently, *ϵ* = *g*(*r*) × *l*(**p**). And the loss becomes:

(8) $$L_{\text{MyLC}}=\text{H}\left({\tilde{\text{q}}}_{\text{MyLC}},\text{p}\right)\text{, where }{\tilde{\text{q}}}_{\text{MyLC}}=(1-\epsilon )\text{q}+\epsilon \text{p}.$$

Algorithm 1 **SMKD: PyTorch-like Pseudocode**

**class** SMKDWithLoss(nn.Module):
    **def** ___init___(self):
        **super** (SMKDWithLoss, self). ___init___ ( )
    **def** forward(self, qA, qB, pA, pB, th):
        # *qA, a refined label from model A*
        # *qB, a refined label from model B*
        # *pA, knowledge/prediction from model A*
        # *pB, knowledge/prediction from model B*
        # *th, the threshold for knowledge selection*
        hpA = torch.sum(-pA * torch.log(pA + 1e-6), 1) *# calculate the entropy of pA*
        hpB = torch.sum(-pB * torch.log(pB + 1e-6), 1) *# calculate the entropy of pB*
        indexB = (hpB < th).nonzero( ) *# select the low entropy sample from model B*
        indexA = (hpA < th).nonzero( ) *# select the low entropy sample from model A*
        lossB2A = cross_entropy(qB[indexB], pA[indexB]) *# distill knowledge from model B to model A*
        lossA2B = cross_entropy(qA[indexA], pB[indexA]) *# distill knowledge from model A to model B*
        lossB2A = sum(lossB2A) / len(indexA) *# average loss over all confident samples from model B*
        lossA2B = sum(lossA2B) / len(indexB) *# average loss over all confident samples from model A*
        **return** lossB2A, lossA2B

### C SMKD for Robust Learning Against Noisy Labels

#### 1) Label Noise Generation

We verify the effectiveness of our proposed SMKD on both synthetic and real-world label noise. For synthetic label noise, we consider symmetric noise and pair-flip noise [16]. For symmetric label noise, a sample’s original label is uniformly changed to one of the other classes with a probability of noise rate r. The noise rates are set to 20%, 40%, 60%, and 80%. For pair-flip noise, the original label is flipped to its adjacent class with noise rates of 20% and 40%, respectively.

#### 2) The Interaction Between SMKD and Self Label modification

As shown in Tables I and II, SMKD, as a new selective MKD method, can be easily combined with existing self training methods as a collaborative mutual enhancer.

In Table I, we explore to train each model using self label modification methods (LS, CP, ProselfLC [2] and MyLC). At the same time, we try four types of knowledge communication: Zero/no knowledge is distilled into the peer model and two models are trained independently; All knowledge is distilled without selection, as SyncMKD does; our proposed methods including SMKD-S and SMKD-P. *Vertically*, from the selective knowledge distillation perspective, we clearly observe that SMKD methods (SMKD-S and SMKD-P) are better than “Zero” and “All” consistently no matter how each model is trained. This empirically demonstrates that selecting confident knowledge for distillation is better. In addition, SMKD-P is slightly better than SMKD-S, mainly due to the fact that a model’s knowledge upgrades and becomes confident as the training progresses.

Table II is an extension of Table I. Results of different noise types and rates are present. Since ProSelfLC and MyLC always performs better than the other approaches, therefore we only apply SMKD over them to explore how much SMKD can enhance stronger baselines.

#### 3) Comparison with Learning with Noisy Labels Methods

In this subsection, our objective is to compare with recent methods for addressing label noise. For simplicity, we only train SMKD-P together with ProSelfLC and MyLC, which are demonstrated to be the best in Section IV-C2. Table III (CIFAR-100) shows results of training ResNet50 on CIFAR-100. SMKD-P+ProSelfLC and SMKD-P+MyLC outperform all the recent label-noise-oriented methods under both pair-flip and symmetric noisy labels. Notably, their improvements are more significant when noise rate rises. We also presents the results on two real-world noisy datasets, Webvision and Food-101 in Table III. For Webvision, we follow the “Mini” setting in [18]. The first 50 classes of the Google resized image subset is treated as training set and evaluate the trained networks on the same 50 classes on the ILSVRC12 validation set. The results of SMKD-P+ProSelfLC and SMKD-P+MyLC are around **5-6%** higher than the latest methods including Co-teaching, APL, CDR, and ProselfLC. Due to the increased difficulty of Food-101, the performance gap across techniques is narrower. SMKD-P+ProSelfLC and SMKD-P+MyLC regularly outperform all compared algorithms.

### D Comparing with Recent MKD Methods

In Table IV, we present the results of the baseline CE, self KD methods (Tf-KD*_reg_* [3], ProselfLC and MyLC), and mutual distillation algorithms (MKD, KDCL, SMKD-P+ProSelfLC, and SMKD-P+MyLC) under noisy scenarios. For self KD methods, we train each model individually (i.e., without mutual distillation) while for MKD methods, we train them together (i.e., with mutual distillation).

- **MKD for two networks of the same architecture**. In Table IV (same), SMKD-P+MyLC achieves **17%-18%** absolute improvement compared to MKD and KDCL. All experiments are trained for 100 epoch.
- **MKD for two networks of different architectures**. In Table IV (difference), we demonstrate SMKD’s effectiveness for training two different networks, ResNet18 and ShufflenetV2. SMKD improves MyLC for around 3% for ResNet18 and 1-3% for ShuffleNetV2. Each experiment is trained for 200 epoch.

### E Hyper-parameters Analysis

#### 1) Analysis of b_2_

Mathematically, according to section III-B, *b*_2_ decides how the knowledge selection threshold changes along with the training epoch *t*. In Figure 3, we fix *η* = 2 and study the effect of *b*_2_ under different noise rates. We observe that the accuracy increases as *b*_2_ decreases for all noise rates. The trend becomes more obvious as the noise rate increases. This empirically verifies the effectiveness of confident knowledge selection again. Furthermore, progressively increasing the confidence criterion leads to better performance. In Figure 4, we further study *b*_2_ under different *η*. The accuracy keeps increasing as *b*_2_ decreases for all *η*. Additionally, the trend is more significant when *η* becomes smaller.

#### 2) Analysis of *η*

As presented in section III-B, *η* is a parameter to linearly scale the knowledge selection criteria. To study *η*, we first analyze the static mode. Table V shows the results of SMKD-S with different *η*. We can see that a lower threshold (i.e., larger *η*) has higher accuracy for all noise rates. This further demonstrates the effectiveness of distilling more confident knowledge. We then analyse the dynamic mode. In Figure 4, the green line (*η* = 4) has the highest accuracy for most *b*_2_ values. Overall, the blue line (*η* = 3) is the second best, while the red line (*η* = 2) has the lowest accuracy. Therefore, we conclude that a smaller *η* is better in both static and progressive modes.

## V Conclusion

We are investigating knowledge selection in MKD and proposing an unified framework for knowledge selection called SMKD. SMKD improves MKD by distilling only confident knowledge to the peer model. Extensive experiments illustrate the effectiveness of SMKD empirically. In addition, our suggested SMKD outperforms comparable MKD algorithms in the presence of label noise and achieves competitive performance in clean circumstances.

## Figures and Tables

**Table I:** The interactions between how each model is trained (i.e., Lable smoothing (LS), Confidence penalty (CP), ProS-elfLC, and our proposed variant MyLC) and what knowledge should be distilled (zero knowledge, all knowledge, and our proposed SMKD-S/P). From each column, we observe the effectiveness of SMKD for MKD (SMKD > ALL > Zero, more detail in Section IV-C). Experiments are done on CIFAR-100 using ResNet34. The symmetric label noise rate is 40%. The average final test accuracies (%) of two models are reported. The performance difference between the two models is negligible.

Distilled Knowledge	LS	CP	ProSelfLC	MyLC
Zero	51.53	51.09	64.07	65.04
All	53.63	53.18	59.26	61.11
SMKD-S(ours)	55.10	53.86	67.26	68.45
SMKD-P(ours)	**56.73**	**56.47**	**68.29**	**69.09**

**Table II:** Results on CIFAR-100 clean test set. All methods use ResNet34 as the network architecture. The top two results of each column are bolded.

Method	Pair-flip label noise					Symmetric label noise		Clean
				
	20%	40%				20%	40%	
CE	63.52	45.40				63.31	47.20	75.58
LS	65.15	50.02				67.45	51.53	76.33
CP	64.97	49.01				65.97	51.09	75.29
Boot-soft	64.04	48.85				63.25	48.41	75.37
ProSelfLC	74.13	69.49				71.49	64.07	75.73
SMKD-S+ProselfLC	75.68	74.22				72.11	67.26	76.25
SMKD-P+ProselfLC	**75.76**	**74.55**				**72.58**	68.29	**77.32**
MyLC	73.12	62.29				71.04	65.04	75.20
SMKD-S+MyLC	75.39	74.32				72.20	**68.45**	75.92
SMKD-P+MyLC	**75.89**	**74.72**				**73.22**	**69.09**	**76.42**

**Table III:** Recent state-of-the-art approaches for label noise are compared. All methods apply ResNet50 as the network architecture. For Food-101, we use a ResNet50 pre-trained on ImageNet. For Webvision, we follow the “Mini” setting in [24], [18], [35], [23]. The top two results of each column are bolded.

Method	CIFAR-100										Real-world noise				
				
	Pair-flip label noise					Symmetric label noise					Food-101				Webvision (Mini)
												
	20%	40%				20%	40%				~20%				~50%
CE	64.10	52.77				63.93	56.82				84.03				57.34
GCE [36]	62.32	55.03				65.62	57.97				84.96				55.62
Co-teaching [16]	58.11	48.46				61.47	53.44				83.73				61.22
Co-teaching+ [17]	56.31	38.03				64.13	55.92				76.89				33.26
Joint [19]	67.35	52.22				54.88	45.64				83.10				47.60
Forward [20]	58.37	39.82				66.12	59.45				85.52				56.33
MentorNet [18]	54.73	45.31				57.27	49.01				81.25				57.66
T-revision [21]	62.69	52.31				64.67	57.15				85.97				60.58
DMI [22]	58.77	42.89				62.77	57.42				85.52				56.93
S2E [37]	58.21	41.74				64.21	43.12				84.97				54.33
APL [23]	59.77	53.25				59.37	51.03				82.17				61.27
CDR [24]	71.93	56.94				68.68	62.72				86.36				61.85
ProSelfLC [2]	73.11	69.49				71.17	60.38				86.97				62.40
SMKD-P+ProselfLC	**75.16**	**73.36**				**73.25**	**64.09**				**87.54**				**67.40**
MyLC	72.25	70.84				69.92	62.80				86.70				64.44
SMKD-P+MyLC	**74.38**	**73.86**				**72.23**	**64.30**				**87.60**				**67.48**

**Table IV:** The performance of SMKD under different settings, two distinct architectures, and the same architectures. SMKD+MyLC outperforms other MKD methods.

	Method	Difference								Same
				
		ResNet18				ShufflenetV2				ResNet34
Baseline	CE	50.63				44.06				47.20
Self KD	Tf-KD_*reg*_ [3]	51.05				44.70				47.39
	ProselfLC [2]	58.51				58.89				64.07
	MyLC	55.94				61.21				65.04
MKD	MKD [8]	60.38				47.72				51.42
	KDCL [11]	55.45				46.10				51.20
	SMKD+MyLC	**68.10**				**64.37**				**69.09**

**Table V:** The results of SMKD-S with different *η*. We train on CIFAR-100 using ResNet-34.

SMKD-S	Symmetric label noise			
	20%	40%	60%	80%
H(u) (*η* = 1)	70.37	59.26	36.18	16.17
1/2 H(u) (*η* = 2)	72.11	65.04	46.15	18.62
1/3 H(u) (*η* = 3)	72.83	66.42	51.34	19.84
1/4 H(u) (*η* = 4)	**73.25**	**67.26**	**54.34**	**22.45**

## Footnotes

1 We remark that the label-noise setting is typical and challenging in real-world machine learning applications, where the given datasets have non-perfect annotations. Additionally, in some recent work, it is shown that the performance gap of different approaches is relatively small in clean scenarios [2], [3], [4], [5]. Therefore, we study selective knowledge distillation and evaluate our design of knowledge selection mainly under the setting of robust deep learning against noisy labels.
